# Predictable Chronic Mild Stress during Adolescence Promotes Fear Memory Extinction in Adulthood

**DOI:** 10.1038/s41598-017-08017-7

**Published:** 2017-08-10

**Authors:** Jia-Hui Deng, Wei Yan, Ying Han, Chen Chen, Shi-Qiu Meng, Cheng-Yu Sun, Ling-Zhi Xu, Yan-Xue Xue, Xue-Jiao Gao, Na Chen, Fei-Long Zhang, Yu-Mei Wang, Jie Shi, Lin Lu

**Affiliations:** 1 National Institute on Drug Dependence and Beijing Key Laboratory of Drug Dependence, Peking University, Beijing, 100191 China; 2 Peking University Sixth Hospital, Peking University Institute of Mental Health, Key Laboratory of Mental Health, Ministry of Health (Peking University), National Clinical Research Center for Mental Disorders (Peking University Sixth Hospital), Peking University, Beijing, 100191 China; 3 School of Basic Medical Sciences, Peking University Health Science Center, Beijing, 100191 China; 4 Peking-Tsinghua Center for Life Sciences and PKU-IDG/McGovern Institute for Brain Research, Peking University, Beijing, 100871 China; 5 Department of Mental Health, First Hospital of Hebei Medical University, Hebei Medical University, Shijiazhuang, 050031 China

## Abstract

Early-life stress in adolescence has a long-lasting influence on brain function in adulthood, and it is mostly recognized as a predisposing factor for mental illnesses, such as anxiety and posttraumatic stress disorder. Previous studies also indicated that adolescent predictable chronic mild stress (PCMS) in early life promotes resilience to depression- and anxiety-like behaviors in adulthood. However, the role of PCMS in associated memory process is still unclear. In the present study, we found that adolescent PCMS facilitated extinction and inhibited fear response in reinstatement and spontaneous recovery tests in adult rats, and this effect was still present 1 week later. PCMS in adolescence increased the activity of brain-derived neurotrophic factor (BDNF)-extracellular signal-regulated kinase 1/2 (ERK1/2) signaling in infralimbic cortex (IL) but not prelimbic cortex in adulthood. Intra-IL infusion of BDNF antibody and the ERK1/2 inhibitor U0126 reversed PCMS-induced enhancement of fear extinction. Moreover, we found that PCMS decreased DNA methylation of the *Bdnf* gene at exons IV and VI and elevated the mRNA levels of *Bdnf* in the IL. Our findings indicate that adolescent PCMS exposure promotes fear memory extinction in adulthood, which reevaluates the traditional notion of adolescent stress.

## Introduction

Excessive fear and anxiety are hallmarks of anxiety disorders, such as posttraumatic stress disorder (PTSD). Understanding the mechanisms of fear inhibition is important for prevention and treatment of fear- and anxiety-related disorders^1^. Extinction of Pavlovian fear conditioning is an extensively used model for studying fear inhibition in rodents and humans^2, 3^. Extinction training does not erase the original fear memories but rather generates a new memory, known as extinction memory, to suppress fear responses^4^. Therefore, the extinguished fear can be spontaneously recovered, renewed, or reinstated^5^. Promoting extinction memory and preventing return of fear response is a prominent strategy to relieve fear-induced anxieties^6^.

Experience during early-life period can produce a long-lasting impact on adult behaviors and neuroendocrine effects^7, 8^. Decades of human and animal studies suggest that stress during adolescence may be a major risk factor for the development and persistence of mental disorders, including PTSD^9–12^. Early life stress and chronic exposure to corticosterone impair fear conditioning and extinction^13, 14^. Chronic unpredictable stress (CUS) during adolescence impairs stress-coping ability and cognitive flexibility^15^. Chronic restrain stress (CRS) slows fear extinction and enhances anxiety-like behavior^16^. On the contrary, predictable chronic mild stress (PCMS), stress exposure within a fixed time window, is reported to improve mood, learning, and memory in adult rats by increasing neurogenesis in the hippocampus^17^. Our previous study also suggested that adolescent PCMS had positive effects on brain functions and enhanced resistance to stress-induced depressive-like behavior in adulthood^18^. However, unknown is the effect of adolescent PCMS experience on fear conditioning and extinction in adulthood.

Medial prefrontal cortex (mPFC) has important roles in modulating the formation, expression and extinction of fear memory^19, 20^, and its different subregions may have distinct functions. Infralimbic cortex (IL) regulates inhibitory memory that underlies extinction and prelimbic cortex (PrL) modulates expression of learned fear^21, 22^. Brain-derived neurotrophic factor (BDNF) participates in many neuronal activity-dependent processes^23, 24^. BDNF knockout in the PrL impaired short- and long-term fear memory but not extinction memory^25^, whereas BDNF in the IL played a critical role in generation and maintenance of extinction memory^26, 27^. Otherwise, BDNF signaling and epigenetic modifications of *Bdnf* are correlated with the early life trauma. BDNF ameliorates CRS-induced impairments in spatial memory^28^ and its expression is inhibited by adolescent CUS exposure^15^. Early maltreatment produces persisting changes in DNA methylation of *Bdnf* ^29^. However, the roles of BDNF and its epigenetic regulation in the adaptive effect of PCMS are still unknown.

In the present study, we investigated whether PCMS exposure during adolescence promotes fear conditioning and extinction in adulthood. The underlying mechanisms concerning BDNF signaling and DNA methylation of *Bdnf* were also explored.

## Results

### PCMS but not CRS nor CUS during adolescence enhanced extinction of fear memory in adulthood

We first investigated whether PCMS, CRS and CUS during adolescence influenced locomotor activities. After 28 days of PCMS, CRS, CUS or handling, locomotor activity was tested. The total distances traveled were analyzed using one-way ANOVA and there was no significant main effect of stress. The Tukey’s *post hoc* test revealed that PCMS, CRS, and CUS had no effects on locomotor activities (all p > 0.05; Supplementary Fig. S1).

**Figure 1:**
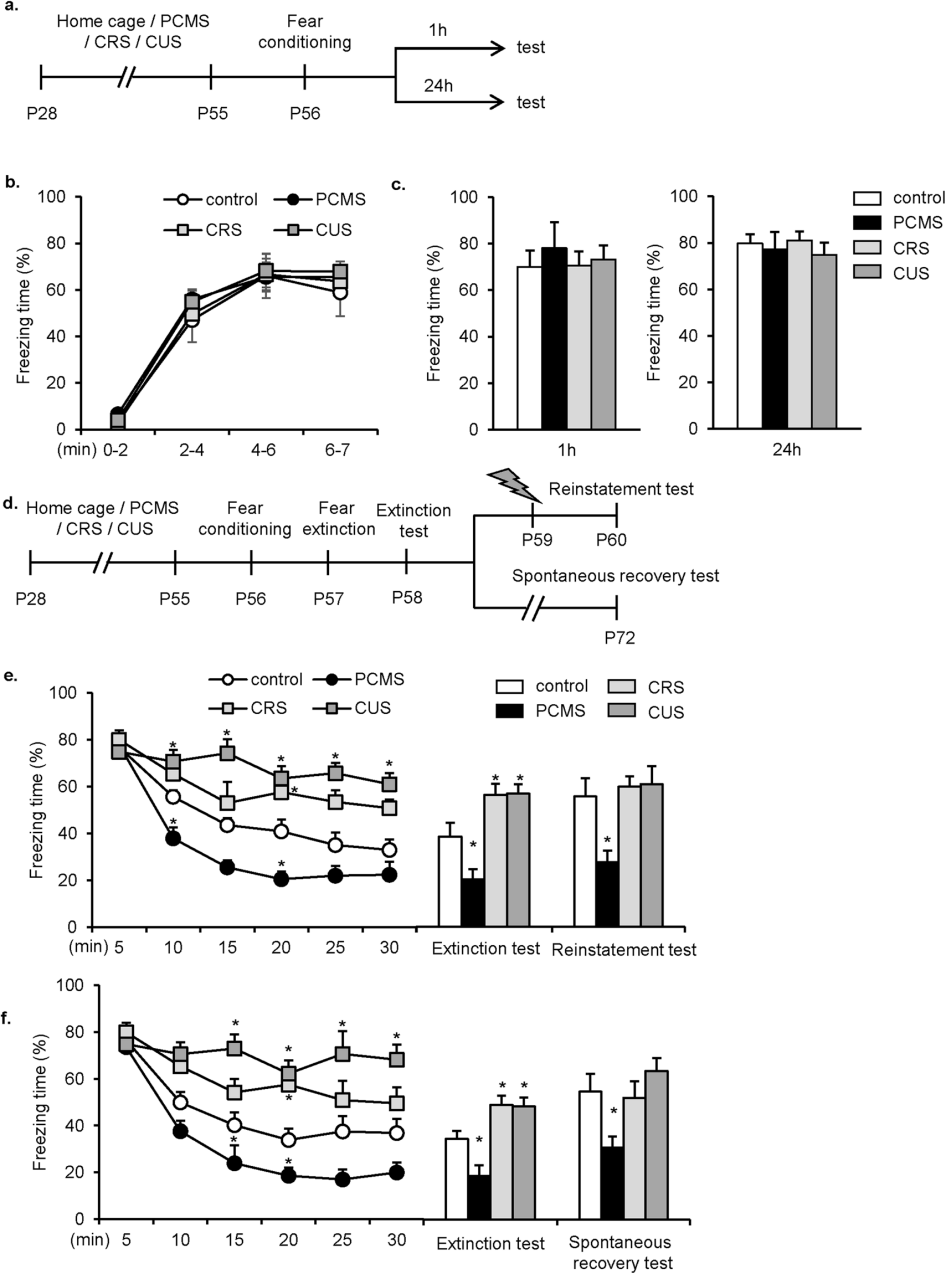
PCMS but not CRS nor CUS during adolescence facilitated the extinction and prevented fear memory return in adulthood. (**a**,**d**) Experimental timeline. (**b**) PCMS, CRS or CUS during adolescence had no significant effect on the conditioning of contextual fear memory (*n* = 8–10 per group). (**c**) PCMS, CRS nor CUS during adolescence had no significant effect on the short term and long term fear memory (*n* = 8–10 per group). (**e**) PCMS exposure during adolescence reduced fear responses in the extinction test and reinstatement test in adulthood (*n* = 8–10 per group). (**f**) PCMS exposure during adolescence reduced fear responses in the extinction test and spontaneous recovery test in adulthood (*n* = 8–10 per group). The data are expressed as the mean ± SEM. *p < 0.05, compared with the control group. PCMS, predictable chronic mild stress; CRS, chronic restraint stress; CUS, chronic unpredictable stress.

Then we investigated whether PCMS, CRS and CUS during adolescence impacted the acquisition, short-term memory (STM) and long-term memory (LTM) of contextual fear conditioning. One day after 28 days of PCMS, CRS, CUS or handling, the rats (*n* = 8–10 per group) underwent contextual fear conditioning (PND56) and were tested 1 h after training, and another four groups of rats (*n* = 8–10 per group) were tested 24 h after training (Fig. 1a). The freezing scores, expressed as the percentage of the freezing time, during conditioning were compared between groups, and repeated-measures ANOVA revealed that PCMS, CRS, and CUS had no effect on the acquisition of fear memory (p > 0.05). Tukey’s *post hoc* test showed there was no difference between groups (all p > 0.05; Fig. 1b). The freezing scores of STM and LTM tests were analyzed using one-way ANOVA and there was no significant main effect of stress. The Tukey’s *post hoc* test revealed that PCMS, CRS, or CUS had no effect on the STM and LTM of contextual fear conditioning, respectively (all p > 0.05; Fig. 1c).

To examine the effects of adolescent PCMS, CUS, and CRS on the extinction of fear memory, the rats (n = 9–10 per group) were subjected to extinction training 24 h after fear conditioning, extinction test 24 h after extinction training and reinstatement test 24 h after footshock **(**Fig. 1d **)**. The freezing scores during extinction training were analyzed using repeated-measures ANOVA, with extinction phase as the within-subjects factor and stress (control, PCMS, CRS, and CUS) as the between-subjects factor. The analysis revealed main effects of stress (*F*_3,32_ = 41.884, *p* < 0.01) and extinction phase (*F*_5,160_ = 35.514, *p* < 0.01) and an extinction phase × stress interaction (*F*_15,160_ = 3.505, *p* < 0.01; Fig. 1e, line graph). Tukey’s *post hoc* test showed that freezing scores decreased in the PCMS group during phases II and IV, increased in the CRS group in phase IV, and increased in the CUS group in phases II-VI (all *p* < 0.05) compared with the control group. For the reinstatement of fear memory, the freezing scores during the extinction test and reinstatement test were analyzed using repeated-measures ANOVA, with test as within-subjects factor and stress (control, PCMS, CRS, and CUS) as the between-subjects factor. This analysis revealed significant effects of test (*F*_1,34_ = 10.341, *p* < 0.01) and stress (*F*_3,34_ = 17.181, *p* < 0.01; Fig. 1e, bar graph) but no test × stress interaction (*p* > 0.05). Tukey’s *post hoc* test revealed that freezing scores were decreased in the PCMS group during the extinction test and reinstatement test (both *p* < 0.05) and increased in the CRS group and CUS group in the extinction test (both *p* < 0.05) compared with the control group. Paired-samples t-test revealed that freezing scores of control rats were increased in reinstatement test compared with those in extinction test (t_9_ = −2.298, p < 0.05), whereas there was no significant difference in freezing scores between reinstatement test and extinction test in PCMS, CRS and CUS groups (all p > 0.05). These results indicate that PCMS in adolescence promoted extinction and prevented the reinstatement of fear memory in adulthood.

To examine the effects of PCMS, CUS, and CRS during adolescence on the spontaneous recovery of fear memory, after extinction training, the rats (*n* = 9–10 per group) were housed in their home cages for 2 weeks before spontaneous recovery test (Fig. 1d). The freezing scores during extinction were analyzed using repeated-measures ANOVA, with test as the within-subjects factor and stress (control, PCMS, CRS, and CUS) as the between-subjects factor. The analysis revealed significant main effects of stress (*F*_3,32_ = 35.204, *p* < 0.01) and extinction phase (*F*_5,160_ = 26.345, *p* < 0.01) and an extinction phase × stress interaction (*F*_15,160_ = 2.954, *p* < 0.01; Fig. 1f, line graph). Tukey’s *post hoc* test showed that freezing scores decreased in the PCMS group during phases III and IV, increased in the CRS group in phase IV, and increased in the CUS group in phases II-VI (all *p* < 0.05), compared with control group. In the extinction test and spontaneous recovery test, the repeated-measures ANOVA analysis revealed significant effects of test (*F*_1,34_ = 7.162, *p* < 0.05) and stress (*F*_3,34_ = 18.041, *p* < 0.01; Fig. 1f, bar graph) but no test × stress interaction (*p* > 0.05). Tukey’s *post hoc* test revealed that freezing scores decreased in the PCMS group during the extinction test and spontaneous recovery test (both *p* < 0.05) and increased in the CRS group and CUS group in the extinction test (both *p* < 0.05) compared with the control group. Paired-samples t-test revealed that freezing scores in control group were significantly increased in spontaneous recovery test compared with those in extinction test (t_9_ = −2.320, p < 0.05), whereas there was no significant difference in freezing scores between spontaneous recovery test and extinction test in PCMS, CRS and CUS groups (all p > 0.05). These results indicate that PCMS in adolescence promoted extinction and blocked the spontaneous recovery of fear memory in adulthood.

Altogether, these results demonstrate that PCMS exposure in adolescence had no effect on the acquisition of fear memory, but enhanced extinction and prevented the return of fear memory in adulthood.

### PCMS during adolescence enhanced the activity of BDNF-ERK1/2 signaling in the mPFC

**Figure 2:**
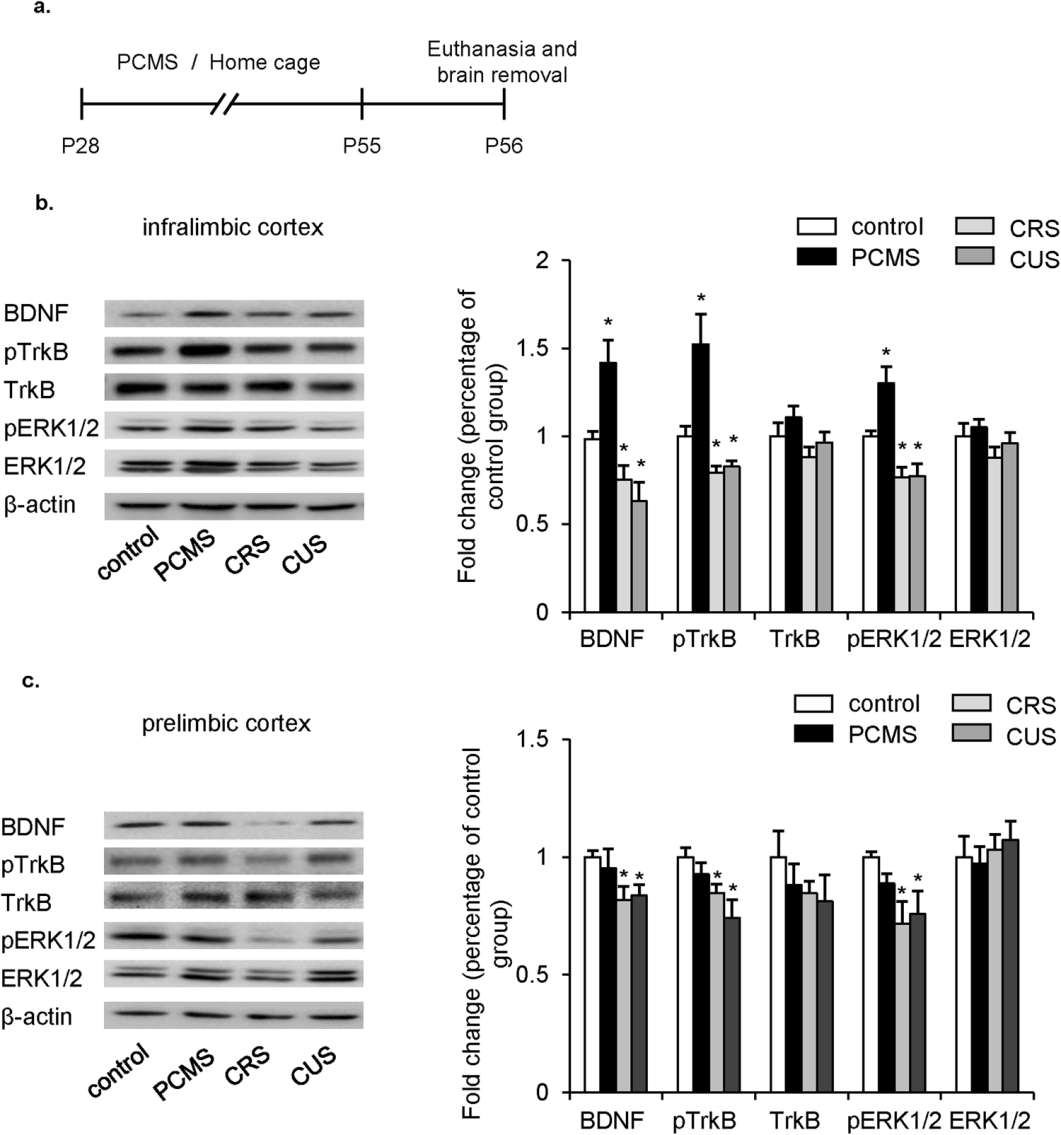
PCMS during adolescence elevated BDNF-ERK1/2 signaling activity in the IL in adulthood. (**a**) Experimental timeline. (**b**,**c**) Protein levels and representative western blot bands of BDNF, pTrkB, TrkB, pERK1/2, and ERK1/2 in the IL (**b**) and PrL (**c**) in different stress manipulated and control rats. Blots shown are cropped from full-length. The data are expressed as the mean ± SEM (*n* = 6 per group). *p < 0.05, compared with the control group.

We examined the effects of PCMS, CRS and CUS in adolescence on the activity of BDNF-ERK1/2 signaling in the mPFC in adulthood. One day after 28 days of PCMS, CRS, CUS or handling, the rats (*n* = 6 per group) were decapitated, and brain tissues were removed and then prepared for subsequent measurements of the levels of BDNF, total TrkB (TrkB), phosphorylated TrkB (pTrkB), total ERK1/2 (ERK1/2), and phosphorylated ERK1/2 (pERK1/2) in the mPFC (Fig. 2a). The western blot data in PrL and IL were analyzed among groups using One-way ANOVA, respectively. In the IL, Tukey’s *post hoc* test showed that, the levels of BDNF (*F*_3,20_ = 11.673, *p* < 0.05), pTrkB (*F*_3,20_ = 10.696, *p* < 0.05), and pERK1/2 (*F*_3,20_ = 15.184, *p* < 0.05) were elevated in PCMS-exposed rats compared with the control group and the opposite trend of these proteins expression were found in CRS- and CUS-exposed rats, whereas no differences were found in the levels of TrkB and ERK1/2 among all groups (Fig. 2b). In the PrL, Tukey’s *post hoc* test showed that, the levels of BDNF (*F*_3,20_ = 4.635, *p* < 0.05), pTrkB (*F*_3,20_ = 4.909, *p* < 0.05), and pERK1/2 (*F*_3,20_ = 3.778, *p* < 0.05) were decreased in CRS- and CUS-exposed rats compared with the control group but no significant changes were found in PCMS-exposed rats. Meanwhile, all of three stress had no effect on the expression of TrkB and ERK1/2 compared with the control group (all *p* > 0.05; Fig. 2c). These results indicate that PCMS during adolescence enhanced the activity of BDNF signaling in the IL.

### PCMS during adolescence caused long-lasting enhancement of fear memory extinction and suppressed reinstatement and spontaneous recovery

**Figure 3:**
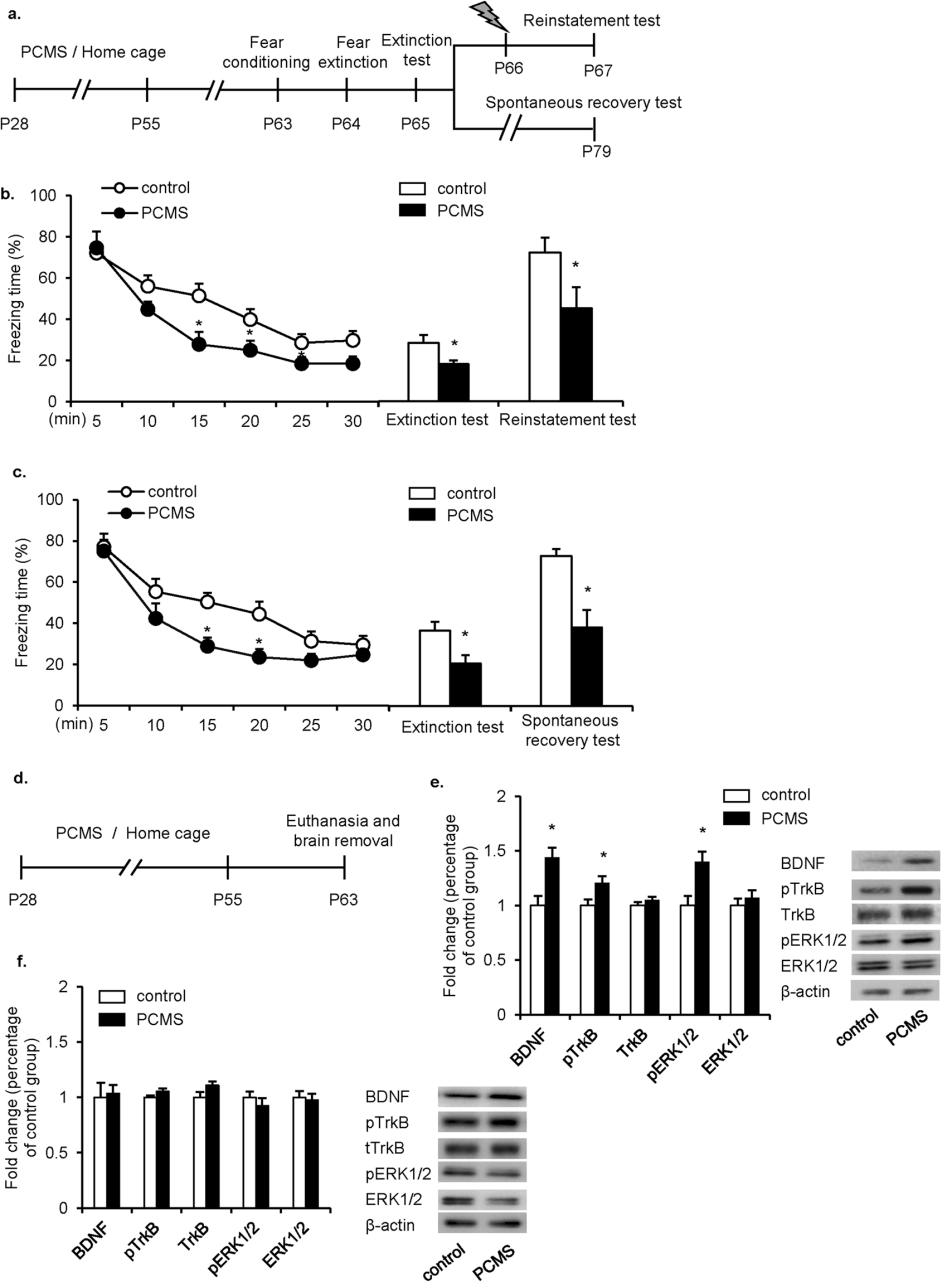
PCMS during adolescence had a prolonged extinction-enhancing effect. (**a**,**d**) Experimental timeline. (**b**,**c**) One week after adolescent PCMS exposure, the rats exhibited an acceleration of fear extinction and lower fear responses in the extinction test, reinstatement test, and spontaneous recovery test. (**b**) Fear responses were reduced in the extinction test and reinstatement test 1 week after PCMS exposure (*n* = 7–9 per group). (**c**) Fear responses in the extinction test and spontaneous recovery test were reduced 1 week after PCMS exposure (*n* = 7–9 per group). (**e**,**f**) Protein levels and representative western blot bands of BDNF, pTrkB, TrkB, pERK1/2, and ERK1/2 in the IL (**e**) and PrL (**f**) in PCMS-exposed and control rats (*n* = 8–10 per group). Blots shown are cropped from full-length. The data are expressed as the mean ± SEM. *p < 0.05, compared with the control group.

We next explored whether PCMS during adolescence has a prolonged effect on fear extinction in adulthood. One week after 28 days of PCMS or handling, the rats (*n* = 7–9 per group) underwent contextual fear conditioning (PND63) followed by extinction training. One day after the extinction test, the rats were given a footshock and tested for reinstatement 24 h later (Fig. 3a). The freezing scores during extinction training were analyzed using repeated-measures ANOVA, with extinction phase as the within-subjects factor and stress (control and PCMS) as the between-subjects factor. This analysis revealed significant main effects of extinction phase (*F*_5,70_ = 43.356, *p* < 0.01), stress (*F*_1,14_ = 6.580, *p* < 0.05) and a stress × extinction phase interaction (*F*_5,70_ = 2.471, *p* < 0.05; Fig. 3b, line graph). Tukey’s *post hoc* test showed that freezing scores decreased in the PCMS group during phases III-V (all *p* < 0.05) compared with control group. In the extinction test and reinstatement test, the repeated-measures ANOVA analysis revealed significant effects of test (*F*_1,14_ = 27.938, *p* < 0.01) and stress (*F*_1,14_ = 18.033, *p* < 0.01) but no test × stress interaction (*F*_1,14_ = 2.516, *p* = 0.135; Fig. 3b, bar graph). Tukey’s *post hoc* test showed that freezing scores decreased in the PCMS group in the extinction test and reinstatement test (both *p* < 0.05). Paired-samples t-test revealed that freezing scores in control group were significantly increased in reinstatement test compared with those in extinction test (t_7_ = −5.523, p < 0.01), whereas in PCMS group, freezing scores in reinstatement test were tendentially but not significantly higher than those in extinction test (t_7_ = −2.363, p = 0.05).

One day after the extinction test, another two groups of rats were housed in their home cages for another 2 weeks and tested for spontaneous recovery (Fig. 3a). The freezing scores during extinction training were analyzed using repeated-measures ANOVA, with extinction phase as the within-subjects factor and stress (control and PCMS) as the between-subjects factor. The analysis revealed significant main effects of extinction phase (*F*_5,70_ = 37.415, *p* < 0.01) and stress (*F*_1,14_ = 5.866, *p* < 0.05) but no stress × extinction phase interaction (*F*_5,70_ = 1.766, *p* = 0.131; Fig. 3c, line graph). Tukey’s *post hoc* test showed that, freezing scores decreased in the PCMS group during phases III and IV compared with control group (both *p* < 0.05). The repeated-measures ANOVA analysis of freezing scores in the extinction test and spontaneous recovery test revealed significant effects of test (*F*_1,13_ = 22.581, *p* < 0.01) and stress (*F*_1,13_ = 29.223, *p* < 0.01) but no test × stress interaction (*F*_1,13_ = 2.756, *p* = 0.121; Fig. 3c, bar graph). Tukey’s *post hoc* test showed, freezing scores decreased in the PCMS group during the extinction test and spontaneous recovery test compared with the control group (both *p* < 0.05). Paired-samples t-test revealed that freezing scores in control group were increased in spontaneous recovery test compared with those in extinction test (t_7_ = −7.143, p < 0.001), whereas there was no significant difference in freezing scores between spontaneous recovery test and extinction test in PCMS group (p > 0.05).

BDNF in the IL may protect individual from perplexity of fear memory^26,^ 27. Above we found that BDNF-pERK1/2 signaling in the IL was activated 1 day after PCMS. We suspected that long-lasting enhancement of fear extinction by PCMS may be mediated by persistent activity of BDNF-ERK1/2 signaling in the IL. Thus, we explored whether PCMS in adolescence has a prolonged effect on the activation of BDNF-ERK1/2 signaling. One week after 28 days of PCMS or handling, the rats (n = 8–10 per group) were decapitated, and brain tissues were collected. The levels of BDNF, TrkB, pTrkB, ERK1/2, and pERK1/2 in the mPFC were measured (Fig. 3d). The western blot data were analyzed using Student’s t-test. In the IL, the levels of BDNF (t_14_ = −2.435, p < 0.05), pTrkB (t_14_ = −2.542, p < 0.05), and pERK1/2 (t_14_ = −3.690, p < 0.05) were elevated in PCMS-exposed rats compared with the control group, whereas no difference was found in the levels of TrkB and ERK1/2 (Fig. 3e). In the PrL. Student’s t-test revealed that the levels of BDNF, TrkB, pTrkB, ERK1/2, and pERK1/2 did not significantly change between groups (all p > 0.05; Fig. 3f).

These results indicate that the enhancement of extinction and suppression of reinstatement and spontaneous recovery of fear memory induced by adolescent PCMS exposure were still present 1 week after PCMS exposure, which may be associated with the activation of BDNF-ERK1/2 signaling in the IL.

### Inhibition of BDNF in the IL prevented the PCMS-induced enhancement of fear extinction

**Figure 4:**
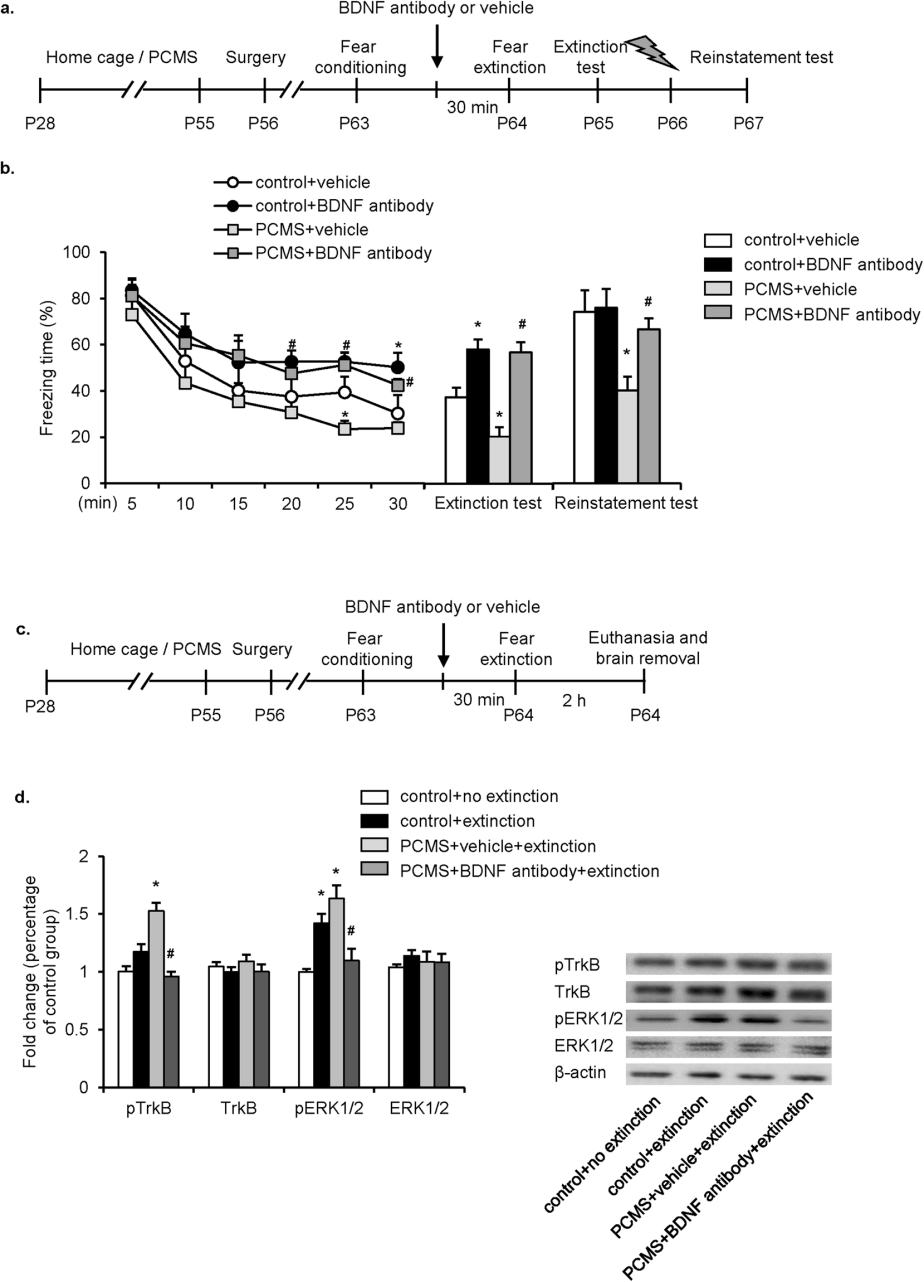
Intra-IL infusion of BDNF antibody reversed the PCMS-induced enhancement of fear extinction. (**a**,**c**) Experimental timeline. (**b**) Intra-IL infusion of BDNF antibody (0.5 μg/0.5 μl per side) blocked the enhancement of extinction and decrease in reinstatement induced by PCMS. The data are expressed as the mean±SEM (*n* = 8–10 per group). *p < 0.05, compared with control+vehicle group; ^#^ *p* < 0.05, compared with PCMS+vehicle group. (**d**) Protein levels and representative western blot bands of pTrkB, TrkB, pERK1/2, and ERK1/2 in the IL after BDNF antibody infusion. Blots shown are cropped from full-length. The data are expressed as the mean±SEM (*n* = 6–8 per group). *p < 0.05, compared with control+no extinction group; ^#^ *p* < 0.05, compared with PCMS+vehicle+extinction group.

BDNF regulated the development of the central nervous system by interacting with TrkB receptors^30^. The inactivation of BDNF-TrkB signaling effectively reversed behavioral changes that were induced by enhanced BDNF expression in the PFC^31^. To investigate the causal role of mPFC BDNF signaling in the effects of adolescent PCMS, we blocked BDNF activity by infusing BDNF antibody in the IL. The rats were assigned to control+vehicle, control+BDNF antibody, PCMS+vehicle, and PCMS+BDNF antibody groups (*n* = 8–10 per group) and underwent stereotaxic surgery after 28 days of PCMS exposure or handling, followed by 7 days of recovery. On PND63, the rats underwent fear conditioning, received an intra-IL infusion of BDNF antibody (0.5 µg/0.5 µl/side) or vehicle (saline) 30 min before fear extinction on the next day, and were subjected to the extinction test 24 h later. The day after the extinction test, the rats were given a footshock and tested for reinstatement 24 h later (Fig. 4a). The freezing scores during extinction training were analyzed using two-way repeated-measures ANOVA, with extinction phase as the within-subjects factor and drug (vehicle and BDNF antibody) and stress (control and PCMS) as the between-subjects factors. This analysis revealed significant effects of extinction phase (*F*_5,140_ = 33.487, *p* < 0.05) and drug (*F*_1,28_ = 10.991, *p* < 0.05, Fig. 4b). No significant effects of stress (*F*_1,28_ = 1.582, *p* > 0.05) and extinction phase×drug interaction (*F*_5,140_ = 1.002, *p* > 0.05) were found, but Tukey’s *post hoc* test revealed that freezing scores were lower in the PCMS+vehicle group than in the control+vehicle group during extinction phases V (*p* < 0.05; Fig. 4b, line graph). Tukey’s *post hoc* test also revealed that compared with the PCMS+vehicle group, the freezing scores in the PCMS+BDNF antibody group increased in phases V and VI (both *p* < 0.05). The analysis of the extinction test and reinstatement test using two-way repeated-measures ANOVA, with test (extinction test and reinstatement test) as the within-subject factors and drug (vehicle and BDNF antibody) and stress (control and PCMS) as the between-subjects factors, revealed significant effects of test (*F*_1,28_ = 17.798, *p* < 0.05), stress (*F*_1,28_ = 8.020, *p* < 0.05), and drug (*F*_1,28_ = 27.275, *p* < 0.05; Fig. 4b, bar graph) and a stress × drug interaction (*F*_1,28_ = 7.283, *p* < 0.05), but no test × stress, test × drug, or test × drug × stress interaction (all *p* > 0.05). In the extinction test and reinstatement test, Tukey’s *post hoc* test revealed freezing scores decreased in PCMS+vehicle group compared with the control+vehicle group (both *p* < 0.05; Fig. 4b, bar graph). However, Tukey’s *post hoc* test also showed that the attenuating effect of adolescent PCMS on freezing scores during the extinction test and reinstatement test were reversed by the intra-IL infusion of BDNF antibody (both *p* < 0.05; Fig. 4b, bar graph). These results indicate that the intra-IL infusion of BDNF antibody reversed the enhancing effect of PCMS on fear extinction.

We then assessed the effects of BDNF inhibition in the IL on the expression of downstream signaling, including pTrkB, TrkB, pERK1/2, and ERK1/2. The rats were assigned to control+no extinction, control+extinction, PCMS+vehicle+extinction, and PCMS+BDNF antibody+extinction groups (*n* = 6–8 per group) and underwent fear conditioning on PND63. The next day, the PCMS+vehicle+extinction and PCMS+BDNF antibody+extinction groups received an intra-IL infusion of saline or BDNF antibody (0.5 µg/0.5 µl/side). Thirty minutes later, the control+extinction, PCMS+vehicle+extinction, and PCMS+BDNF antibody+extinction groups were subjected to extinction training. Two hours later, brain tissues were collected from the four groups (Fig. 4c). The western blot data were analyzed among groups using One-way ANOVA. There were significant effects on expression of pTrkB (*F*_3,27_ = 3.349, *p* < 0.05) and pERK1/2 (*F*_3.27_ = 10.359, *p* < 0.05). Tukey’s *post hoc* test revealed that, the extinction of conditioned fear was accompanied by a significant increase in pERK1/2 (*p* < 0.05; Fig. 4d) but not pTrkB, TrkB, or ERK1/2. Tukey’s *post hoc* test also showed that compared with the control+no extinction group, the levels of pTrkB and pERK1/2 increased in the PCMS+vehicle+extinction group, which were reversed by an intra-IL infusion of BDNF antibody (all *p* < 0.05; Fig. 4d). Student’s t-test revealed that infusion of BDNF antibody into IL decreased levels of pTrkB and pERK1/2 compared with PCMS+vehicle+extinction group (pTrkB: t10 = 2.454, p < 0.05; pERK1/2: t10 = 2.466, p < 0.05; Supplementary Fig. S3). These results indicate that BDNF signaling is involved in the enhancing effect of adolescent PCMS on fear extinction in adulthood.

### Inhibition of ERK1/2 in the IL reversed the PCMS-induced enhancement of fear extinction

**Figure 5:**
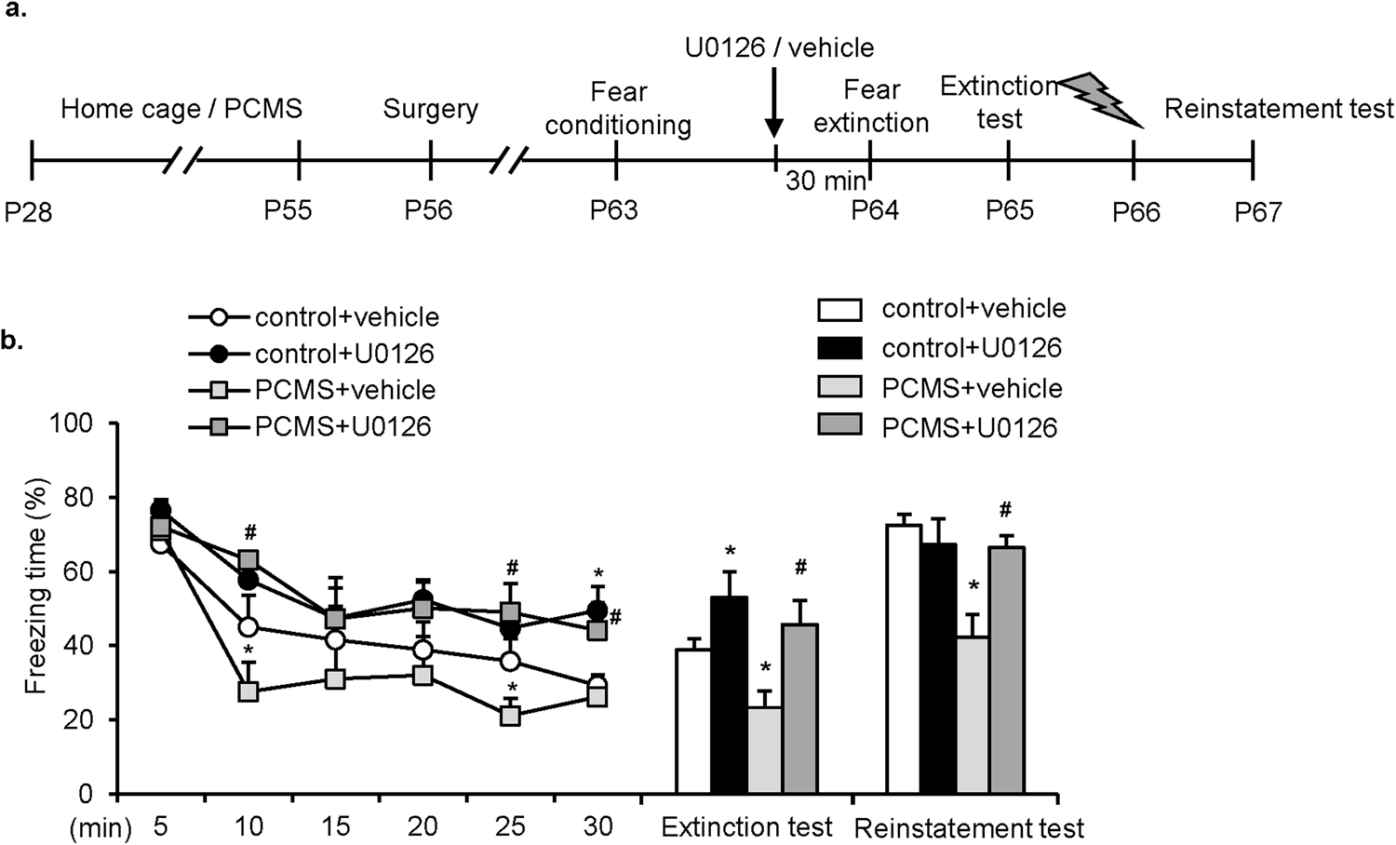
Intra-IL infusion of the ERK1/2 inhibitor U0126 reversed the PCMS-induced enhancement of fear extinction. (**a**) Experimental timeline. (**b**) U0126 blocked the effect of PCMS on extinction, extinction retention, and reinstatement. The data are expressed as the mean±SEM (*n* = 8–10 per group). *p < 0.05, compared with control+vehicle group; ^#^ *p* < 0.05, compared with PCMS+vehicle group.

BDNF binds to TrkB receptors, triggering the activation of ERK1/2. The BDNF-ERK1/2 signaling pathway is crucial for the retention of fear memory^32^. To investigate the function of BDNF-ERK1/2 signaling in the IL in the enhancing effect of PCMS on fear extinction, we assigned rats to control+vehicle, control+U0126, PCMS+vehicle, and PCMS+U0126 groups and infused U0126, an inhibitor of ERK1/2 phosphorylation^33^, or vehicle (20% DMSO) in the IL 30 min before extinction training (Fig. 5a). The freezing scores during extinction training were analyzed using two-way repeated-measures ANOVA, with extinction phase as the within-subjects factor and stress (control and PCMS) and drug (vehicle and U0126) as the between-subjects factors. This analysis revealed significant effects of extinction phase (*F*_5,150_ = 12.239, *p* < 0.05) and drug (*F*_1,30_ = 20.437, *p* < 0.05) and an extinction phase × drug interaction (*F*_5,150_ = 2.616, *p* < 0.05; Fig. 5b, line graph). No significant main effect of stress was found (*F*_1,30_ = 3.956, *p* = 0.056). Tukey’s *post hoc* test revealed that freezing scores were higher in the PCMS+U0126 group than in the PCMS+vehicle group during extinction phases II and V-VI (both *p* < 0.05). Tukey’s *post hoc* test also showed that compared with the control+vehicle group, freezing scores significantly decreased in the PCMS+vehicle group in extinction phases II and V (*p* < 0.05). The analysis of the extinction test and reinstatement test using two way repeated-measures ANOVA, with test (extinction test and reinstatement test) as the within-subjects factor and drug (vehicle and U0126) and stress (control and PCMS) as the between-subjects factors, revealed significant effects of test (*F*_1,30_ = 30.051, *p* < 0.05), stress (*F*_1,30_ = 9.116, *p* < 0.05), and drug (*F*_1,30_ = 16.692, *p* < 0.05) and a stress×drug interaction (*F*_1,30_ = 4.240, *p* < 0.05; Fig. 5b, bar graph), but no test × stress, test × drug, or test × drug × stress interaction (all *p* > 0.05). Tukey’s *post hoc* test revealed that in the extinction test and reinstatement test, freezing scores decreased in the PCMS+vehicle group compared with the control+vehicle group (both *p* < 0.05). The attenuating effect of adolescent PCMS on freezing scores during the extinction test and reinstatement test were reversed by the intra-IL infusion of U0126 (both *p* < 0.05). These results indicate that inhibiting the activity of ERK1/2 in the IL reverses the effect of PCMS on fear extinction.

### PCMS during adolescence decreased methylation modification of the *Bdnf* gene

**Figure 6:**
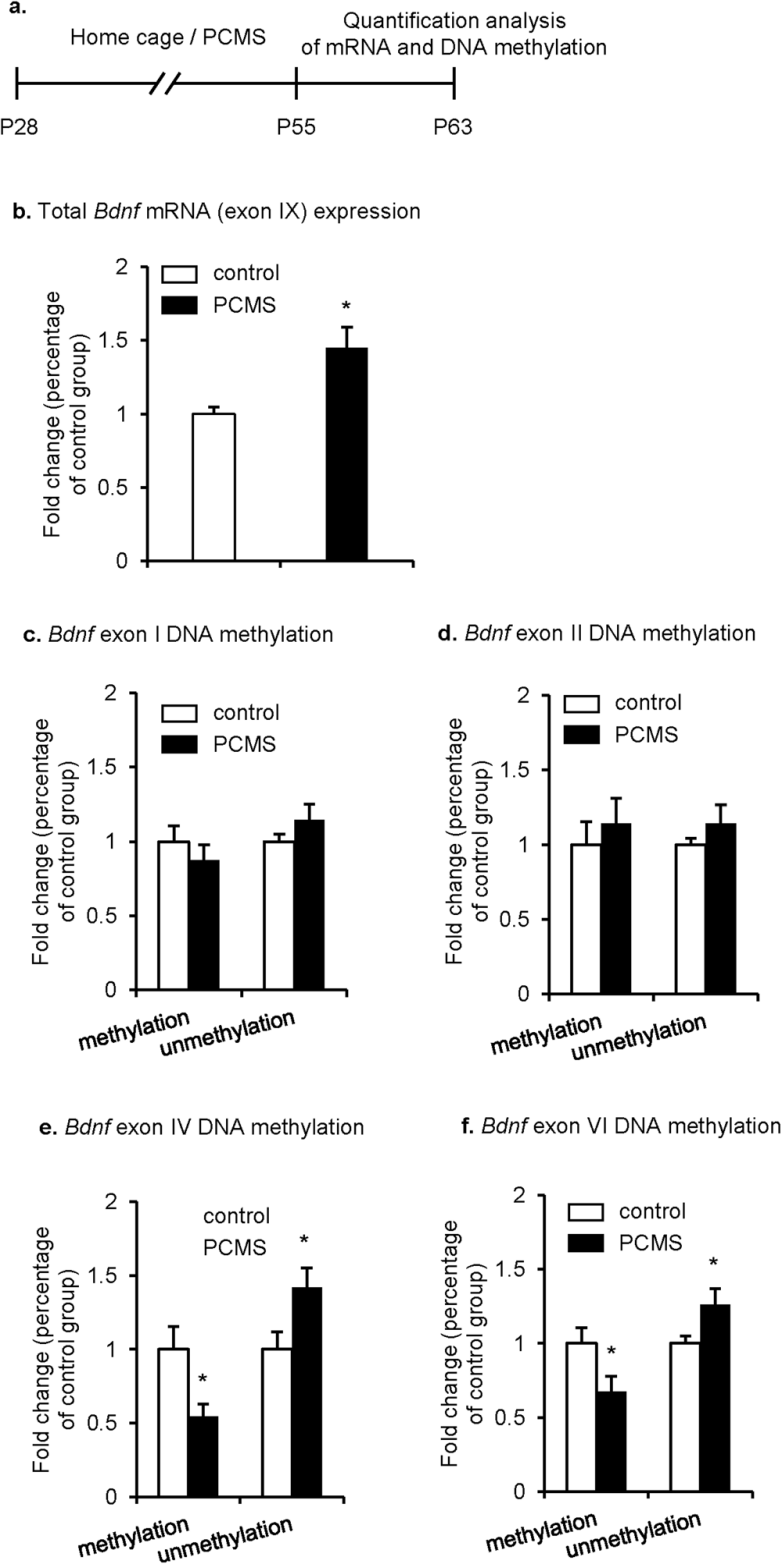
PCMS during adolescence increased *Bdnf* gene transcription and decreased the level of *Bdnf* DNA methylation in the IL. (**a**) Experimental timeline. (**b**) Total mRNA levels of *Bdnf* in the IL. (**c**–**f**) Levels of DNA methylation and unmethylation of *Bdnf* at exons I, II, IV, and VI in the IL. The data are expressed as the mean±SEM (*n* = 10 per group). *p < 0.05, compared with the control group.

Methylation status is long lasting^34^ and DNA methylation regulates the transcription of several genes that are responsible for stress response^35^. Thus, we assessed whether PCMS exposure triggered prolonged changes in the DNA methylation of *Bdnf*. We first examined total mRNA levels of *Bdnf* at exon IX in the IL one week after rats were exposed to 28 days of PCMS during adolescence (Fig. 6a). Student’s *t*-test revealed that the levels of *Bdnf* mRNA in PCMS-exposed rats were significantly elevated in the IL compared with the control group (*t*_18_ = −3.082, *p* < 0.05; Fig. 6b). These results indicate that *Bdnf* gene transcription increased in the IL after PCMS exposure during adolescence.

The *Bdnf* gene has multiple initiation sites that allow isoform-specific mRNA transcription^36^. We investigated whether PCMS-induced alterations in *Bdnf* gene transcription were correlated with *Bdnf* DNA methylation. The analysis of the methylation-specific real-time PCR data using Student’s *t*-test showed that PCMS exposure during adolescence decreased the levels of methylated DNA at exon IV (*t*_18_ = 2.577, *p* < 0.05; Fig. 6e) and exon VI (*t*_18_ = 2.245, *p* < 0.05; Fig. 6f) and increased the levels of unmethylated DNA at exon IV (*t*_18_ = −2.327, *p* < 0.05; Fig. 6e) and exon VI (*t*_18_ = −2.252, *p* < 0.05; Fig. 6f). Student’s t-test showed that no significant changes in the levels of methylated and unmethylated DNA at exons I and II were found after PCMS exposure (all *p* > 0.05; Fig. 6c,d). These findings showed that PCMS during adolescence decreased the level of DNA methylation of *Bdnf* and increased *Bdnf* gene transcription in the adult IL.

## Discussion

In the present study, we evaluated the effects of early-life PCMS exposure on fear conditioning and extinction. PCMS exposure during adolescence accelerated extinction and inhibited fear return in adulthood, while CUS and CRS suppressed extinction. The extinction-enhancement effect of PCMS was present 1 week after PCMS. PCMS increased the activity of BDNF-ERK1/2 signaling in IL. An intra-IL infusion of BDNF antibody or ERK1/2 inhibitor reversed the enhancing effect of adolescent PCMS on fear extinction in adulthood. Lastly, we found that PCMS exposure in adolescence decreased the levels of DNA methylation of *Bdnf* at exons IV and VI. Altogether, our results indicate that PCMS during adolescence promoted fear extinction and prevented fear return, which may be mediated by the BDNF-ERK1/2 signaling in IL.

Early experiences and experience-related neural changes play pivotal roles in emotion and cognition in adulthood^37^. In the present study, we found that PCMS exposure during adolescence enhanced fear extinction, and inhibited the return of fear memory. The results suggested that adolescent PCMS, which is a predictable and much milder stress, can produce a potentially adaptive advantage in adult stressful environments. The results were consistent with our previous study that PCMS during adolescence produced antidepressant- and anxiolytic-like effects and enhanced the abilities to resist depressive- and anxiety-like behaviors by chronic unpredictable stress (CUS) in adulthood^18^. Parihar *et al*. found PCMS during adulthood also decreased depressive- and anxiety-like behaviors and enhanced memory function in Morris water maze and novel object recognition, indicating that the protective effects of PCMS might not be specific for adolescence. However, adolescence, transiting from childhood to adulthood^7^, is a critical period of brain maturation, during which the brain undergo extensive morphological and functional remodeling^38^. Compared with adulthood, the stage of brain development in adolescence was vulnerable to environmental influence and experience^38, 39^. Stress in this brain-developing period could produce long-lasting impact on brain function while the effects of stress on adult brain were transient^39, 40^. It was also supported by our result that the extinction-enhancement effect of PCMS in adolescence can persist for at least one week. Overall, we speculate that PCMS in adolescence may affect brain development and maturation, thus producing long-lasting effect on behaviors in adulthood.

In this study, we found that PCMS exposure during adolescence accelerated extinction, while CUS and CRS suppressed extinction. This effect was stress paradigm-specific. The characteristics of the stressor include controllability, predictability, ambiguity, chronicity, and intermittence^41^. These specific characteristics of stressors are important factors in determining whether early life experiences ultimately produce protective or deleterious effect^42^. Prior chronic unpredictable and high strength events, including chronic unpredictable stress (CUS) and chronic restraint stress (CRS), usually lead to negative effects in later life. CUS during adolescence decreased BDNF-ERK1/2 signaling in mPFC and impaired stress-coping ability^15^. It also resulted in spatial memory impairment^43^. CRS during adolescence decreased hippocampal neurogenesis, leading to impairments in hippocampus-dependent fear memory^44^. CRS also increased anxiety-like behaviors and impaired the acquisition and retrieval of fear extinction^16, 45–48^. Chronic social-isolated adolescent rats showed significant deficits in the extinction of conditioned fear with the mechanism involving noradrenergic signaling^48^. CRS impaired acquisition of fear extinction in adolescent rats but not adult rats^45^. Consistent with these studies, we found that CUS and CRS suppressed the extinction of conditioned fear memory. In these cases, excessive intensity and duration of the stressor exceeded endurance, inducing aberrant adaptation. In contrast, some controllable and mild stressors help adolescents develop adaptive stress responses and become resilient to negative stressor in future^17, 18, 49^. For example, intermittent mother-offspring separation and PCMS relieved anxiety^18, 50^. We found that PCMS in adolescence can facilitate extinction and prevent fear return in the present study. However, the protective and adaptive roles of specific stress and the underlying mechanisms need to be further investigated. Collectively, our results compelled a reevaluation of the traditional notion that early stress predicts adverse outcomes in the future.

For the mechanisms underlying the protective effect of PCMS, a previous study showed that PCMS moderately increased corticosterone concentrations for shorter durations, which facilitated beneficial responses in the brain^17^. Mild stress or lower doses of corticosterone or dexamethasone induced transient increases in the levels of neurotrophic factors, such as BDNF, insulin-like growth factor-1, and fibroblast growth factor-2^51, 52^. Moreover, animals with early stress that exhibited enhanced BDNF levels had improved performance in the Morris water maze^42^. In the present study, rats that were exposed to PCMS in adolescence exhibited higher levels of BDNF, indicating that adolescent PCMS exposure might closely resemble the effects of low-dose corticosterone exposure. Besides, our previous study demonstrated that adolescent PCMS had positive effects on brain functions and promoted the resistance to stress in adulthood, and mPFC is critical for these adaptive changes^18^. In this study, PCMS exposure in adolescence increased the levels of BDNF, pTrkB, and pERK1/2 in IL, while CUS and CRS decreased levels of BDNF, pTrkB and pERK1/2 in both IL and PrL. The enhancement of extinction memory induced by adolescent PCMS exposure was offset by the inhibition of BDNF or ERK1/2 in IL. These results were consistent with previous studies that fear extinction requires BDNF in IL but not PrL^26, 27^ and requires the activation of ERK1/2 signaling^53–55^. CUS exposure during adolescence inhibited BDNF- ERK1/2 signaling in adult mPFC, which was consistent with our present study^15^. Besides, previous studies reported that dendritic morphology in mPFC was changed by chronic stress, e.g. CRS decreased the spine density of pyramidal neurons in mPFC^56, 57^. BDNF overexpression could increase spine density in hippocampus and amygdala^58, 59^. These finding indicate that BDNF-ERK1/2 signaling in IL plays a vital role in the extinction-enhancing effect of adolescent PCMS exposure.

DNA methylation is an epigenetic modification, regulating gene expression by adding a methyl group at the C5 position of cytosine to form 5-methylcytosine, and plays a critical role in gene silencing through chromatin remodeling^60^. The methylation changes were enduring^11^. Early neglect or trauma can epigenetically program the stress system, leading to aberrant regulation of the HPA axis and maladaptive, prolonged responses to stressors encountered in later life^11, 35, 61, 62^. Increases in BDNF were shown to be associated with a decrease in methylation of the *Bdnf* gene^63, 64^. Epigenetic modifications of *Bdnf* were related to memory, stress, and neuropsychiatric disorders^64^. The predator exposure significantly increased *Bdnf* DNA methylation in the dorsal hippocampus^65^. Maternal separation decreased repressive histone methylation of the *Bdnf* exon IV promoter and enhanced BDNF levels^42^. In the present study, an increase in the level of *Bdnf* mRNA and decrease in the levels of *Bdnf* methylation at exons IV and VI were found in adolescent PCMS-exposed rats, which might influence the persisting BDNF expression and brain functions in adulthood^29, 66^. Our results were consistent with previous findings that *Bdnf* exon IV is regulated by fear memory and early life adversity^29, 35^. Histone methylation at the *Bdnf* IV promoter was decreased in maternal separated rats with improved performance in the Morris water maze in adulthood^42^. With regard to previous findings that BDNF in IL is involving in generation and maintainance of extinction memory^26, 27^, we speculated that epigenetic regulation of BDNF expression in IL is crucial for the enhancement effect of adolescent PCMS exposure on fear extinction in adulthood.

In conclusion, our results indicate that PCMS exposure during adolescence promoted fear extinction and inhibited return of fear memory in adulthood, while CUS and CRS suppressed extinction. BDNF-related signaling pathways and epigenetic modification of the *Bdnf* gene may be involved in the enhancement of fear extinction by adolescent PCMS exposure. Our results support the hypothesis that specific predictable mild stress exposure during adolescence may ameliorate fear-related disorders.

## Materials and Methods

### Animals

All of the experiments were performed in accordance with the National Institutes of Health Guide for the Care and Use of Laboratory Animals and were approved by the Biomedical Ethics Committee for Animal Use and Protection of Peking University. Three hundred and sixty-one male Sprague Dawley rats, 21 days old and weighing 45–55 g upon arrival, were obtained from the Laboratory Animal Center, Peking University Health Science Center. The animals were allowed to acclimate for 7 days before stress exposure. All of the rats were housed under standard laboratory conditions with controlled room temperature (23 ± 2 °C) and humidity (50 ± 5%) and free access to food and water under a 12 h/12 h light/dark cycle.

### Predictable chronic mild stress

The PCMS protocol was conducted according to previous studies^17, 18^. Beginning on postnatal day 28 (PND28), the rats were guided into stainless-steel cylinders that had a ventilation grille on the head side and sliding door on the tail side. They remained inside the cylinder for 5 min daily, during which they were unable to move. They were then returned to their home cages. The size of the restraint cylinder was adjusted to the growth of rats. The stress paradigm was conducted for 28 continuous days at the same time each day (between 3:00 PM and 5:00 PM) to ensure that the time of the stress was predictable. Animals in the control group received no stress but were handled every day.

### Chronic unpredictable stress

The chronic unpredictable stress (CUS) protocol was based on previous studies^18, 67, 68^. A total of 12 different stressors were used, including cold for 1 h at 4 °C, restraint for 1 h, food deprivation for 24 h, water deprivation for 24 h, light/dark cycle reversal for 36 h, vibration for 1 h, tilting cages (45°) for 24 h, forced cold swim for 5 min, crowding for 24 h, no bedding for 24 h, soiled bedding for 24 h, and tail clamp for 1 min. Beginning on PND28, each rat was exposed to two of these stressors each day for 28 continuous days. The time at which the stressors were applied was unpredictable.

### Chronic restraint stress

The chronic restraint stress (CRS) protocol was conducted as previously described^18^. Beginning on PND28, each rat was fixed in a stainless-steel cylinder (the same one used for PCMS), where it remained immobile for 2 h daily (3:00 PM to 5:00 PM) for 28 continuous days.

### Contextual fear conditioning

Contextual fear conditioning was conducted using a video-based fear conditioning system (Beijing Macro Ambition S&T Development Co., Ltd, Beijing, China) as previously reported^69, 70^. All of the procedures were conducted in conditioning chambers (30 cm × 30 cm × 50 cm). The walls were constructed with black polyvinyl chloride and the floor was constructed with stainless steel rods (0.5 cm diameter, 1.0 cm apart) that were used to deliver foot shocks. Each conditioning chamber was enclosed in an acoustic isolation box. A dim light illuminated the chamber during the procedures.

#### Conditioning

On the training day, the rats were placed in the conditioning chamber and allowed to explore it for 2 min, after which they received three electric footshocks (0.8 mA, 1 s) at 2-min intervals. After the last shock, the rats were allowed to explore the conditioning chamber for an additional 1 min before being returned to their home cages. The chambers were cleaned with 75% alcohol to eliminate any residual odor before placing the rats in them.

#### Extinction

During extinction, the rats were exposed to the conditioning chamber for 30 min without footshock. The 30-min extinction training was divided into six phases (phase I: 0–5 min, phase II: 6–10 min, phase III: 11–15 min, phase IV: 16–20 min, phase V: 21–25 min, and phase VI: 26–30 min).

#### Test

All memory tests were conducted by exposing the rats to the conditioning chamber without footshock for 5 min. The extinction test was performed 24 h after extinction. In reinstatement experiment, the rats received a footshock (0.8 mA, 1 s) in another context 24 h after the extinction test. The reinstatement test was conducted 24 h after the footshock. The spontaneous recovery test was performed in another group of rats 2 weeks after the extinction test.

#### Freezing

Freezing behavior was video-recorded and offline analyzed using JLbehv-LAG-4 software (Shanghai Jiliang Software Technology Co. Ltd, Shanghai, China). The freezing score is expressed as the percentage of the time spent freezing during the test. Freezing behavior was defined as the lack of all movements, with the exception of respiration.

### Locomotor Activity Test

Locomotor Activity Test was conducted based on previous studies^69, 71^. Locomotor activity was measured with an automated video tracking system (DigBehv-LM4; Shanghai Jiliang Software Technology, Shanghai, China) that contained clear Plexiglas chambers (40 cm*40 cm*65 cm). Vdeo files were analyzed using DigBehv analysis software. Locomotor activity is expressed as the total distance traveled in centimeters during a predetermined period of time (in 30 min).

### Surgery

The rats (weighing 270–290 g) were anesthetized with sodium pentobarbital (50 mg/kg, i.p.). Stainless-steel guide cannulas (22 gauge) were bilaterally implanted in the IL (anterior/posterior, +2.9 mm; medial/lateral, ±2.3 mm; dorsal/ventral, −4.8 mm)^72–74,^. The cannulas were placed at a 16° angle toward the midline to avoid penetration of the lateral ventricle. The cannulas were anchored to the skull with screws and dental cement. After surgery, the rats were single-housed and allowed 7 days to recover, during which they were handled every day until fear conditioning.

### Intracranial drug infusions and systemic treatment

BDNF antibody (lyophilized powder; Millipore, Bedford, MA, USA) was dissolved in saline and intracranially administered at a dose of 0.5 µg/0.5 µl/side based on a previous study^75^. U0126 (Sigma-Aldrich, St. Louis, MO, USA), an inhibitor of extracellular signal-regulated kinase 1/2 (ERK1/2), was dissolved in 20% dimethylsulfoxide (DMSO) and intracranially administered (100 ng/0.5 µl/side) according to our previous studies^76, 77^. For intracranial drug infusions, the drugs were infused bilaterally over 2 min, and the needle was kept in place for an additional 1 min to allow drug diffusion.

### Histology

The cannula placements were confirmed in 30-μm-thick sections using Nissl staining under light microscopy (Supplementary Fig. S2). Five rats with misplaced cannulas were excluded from the statistical analysis. Indian ink (0.5 µl/side) was infused bilaterally into IL to confirm the diffusion route (Supplementary Fig. S2).

### Western blot assays

The rats were decapitated 1 or 7 days after 28 days of PCMS exposure or handling (control), or 2 h after fear extinction training, and the protocol of tissue preparing and western blot was based on our previous study^6, 72, 78, 79^. The brains were rapidly extracted and frozen in dry ice. The brains were stored at −80 °C. Bilateral tissue punches (12 gauge) of the PrL and IL were lysed in RIPA lysis buffer (Applygen Technology, Beijing, China) with protease inhibitor and phosphatase inhibitor mixture for 30 min. They were then homogenized (15 s × 3, 5 s intervals) with an electrical disperser (Wiggenhauser, Sdn Bhd). The tissue homogenates were subjected to 10,000 × *g* centrifugation at 4 °C for 15 min. The protein concentrations of all of the samples were determined using the bicinchoninic acid (BCA) assay kit (Beyotime Biotechnology, Shanghai, China). All of the samples were further diluted in RIPA lysis buffer to normalize the protein concentrations. Five × loading buffer (16% glycerol, 20% mercaptoethanol, 2% sodium dodecyl sulfate [SDS], 0.05% bromophenol blue) was added to each sample (4:1, sample: loading buffer) before boiling for 5 min. The samples were stored at −80 °C until analysis. The samples were subjected to SDS-polyacrylamide gel electrophoresis (10% acrylamide/0.27% *N*,*N’*-methylenebisacrylamide resolving gel) for approximately 40 min at 80 V in stacking gel and approximately 1 h at 120 V in resolving gel. Proteins were electrophoretically transferred to polyvinylidene fluoride membranes (Millipore, Bedford, MA, USA) at 250 mA for 1–3 h. Membranes were washed with Tris-buffered saline plus 0.05% Tween-20 (TBST, pH 7.4) and then incubated in blocking buffer (5% bovine serum albumin in TBST) overnight at 4 °C. The next day, the membranes were incubated for 45 min at room temperature on a shaker with anti-BDNF antibody (1:1000, Abcam, Cambridge, UK), anti-tropomyosin-related kinase B (TrkB) antibody (1:1000, Abcam, Cambridge, UK), anti-pTrkB antibody (1:1000, Abcam, Cambridge, UK), anti-ERK1/2 antibody (1:1000, Cell Signaling Technology, MA, USA), anti-pERK1/2 antibody (1:1000, Cell Signaling Technology, MA, USA), or anti-β-actin antibody (1:2000, Sigma-Aldrich, St. Louis, MO, USA) in TBST plus 5% bovine serum albumin and 0.05% sodium azide. After four 5-min washes in TBST buffer, the blots were incubated for 45–55 min at room temperature on a shaker with horseradish peroxidase (HRP)-conjugated secondary antibody (goat anti-rabbit immunoglobulin G for BDNF, TrkB, pTrkB, ERK1/2, pERK1/2; goat anti-mouse for β-actin; Santa Cruz Biotechnology, Santa Cruz, CA, USA) diluted 1:5000 in blocking buffer. The blots then underwent four 5-min washes with TBST, and immunostaining was visualized using the EZ-ECL chemiluminescence detection kit. The immunoblots were quantified with the Gel Doct EZ system (Bio-Rad, Hercules, CA, USA). Band intensities for BDNF, TrkB, pTrkB, ERK1/2, and pERK1/2 were normalized to β-actin and analyzed using Quantity One 4.4.0 software (Bio-Rad, Hercules, CA, USA).

### Measuring mRNA levels by RT-PCR

The protocol was based on our previous study^71^. The preparation of brain tissues from the IL was the same as western blot assay, and total RNA was extracted using the PureLink RNA Micro Kit (Invitrogen, Carlsbad, CA, USA) according to the manufacturer’s instructions. The purified RNA samples were stored at −80 °C until the RT-PCR assay. Reverse transcription was performed using the PrimeScript RT reagent Kit (TakaRa, otsu,Japan) and SYBR select master mix (Invitrogen, Carlsbad, CA, USA). Genomic DNA removal was performed using the CFX96 real-time PCR system (Bio-Rad, Hercules, CA, USA) at 42 °C for 2 min. cDNA amplification was performed at 95 °C for 30 s, followed by 40 cycles of 95 °C for 5 s and 60 °C for 30 s and incubation at 70 °C for 10 min. β-Tubulin-4 quantification was used as an internal control to normalize mRNA levels. The primers for *Bdnf* mRNA at exon IX and β-tubulin-4 mRNA were synthesized according to a previous study^29^.

### Methylation-specific real-time PCR analysis

The protocol was based on our previous study^71^. DNA was isolated from brain tissues of the IL and purified using the TIANamp Micro DNA Kit (Tiangen, Beijing, China) according to the manufacturer’s instructions. Methylation levels were assessed using methylation-specific real-time PCR. The DNA was processed for bisulfite modification using the BisulFlash DNA modification kit (Eptigentek, Farmingdale, NY, USA). Quantitative RT-PCR was used to examine the DNA methylation of *Bdnf*. *β-Tubulin-4* quantification was used as an internal control to normalize the DNA methylation levels. The methylated and unmethylated primers for *Bdnf* at exons I, II, IV, and VI and *β-Tubulin-4* were based on a previous study^29^. The comparative cycle threshold method was used to calculate differences in gene expression between samples. DNA methylation status in different manipulation groups is presented as fold changes relative to the naive group.

### Statistical analysis

All of the statistical analyses were performed using SPSS 17.0 software (SPSS, Chicago, IL, USA). The data are expressed as mean ± standard error of mean (SEM) and were analyzed using analysis of variance (ANOVA) with appropriate between- and within-subjects factors or using the paired-samples *t*-test or student’s *t*-test (see Results for details). Significant main effects and interactions (*p* < 0.05, two-tailed) in the factorial ANOVAs were further analyzed using Tukey’s *post hoc* test as appropriate. Values of *p* < 0.05 were considered statistically significant.

### Data Availability

The datasets generated and analyzed during the current study are available from the corresponding author on reasonable request.

## Electronic supplementary material

Supplementary Figures
