# Supplementary material for: Predictable Chronic Mild Stress during Adolescence Promotes Fear Memory Extinction in Adulthood
Source: Sci Rep. 2017 Aug 10;7:7857. doi: 10.1038/s41598-017-08017-7 (PMC5552791; doi:10.1038/s41598-017-08017-7)
Supplement: Supplementary file 1 — Supplementary Figures [file 41598_2017_8017_MOESM1_ESM.pdf]

June 16, 2017, the revised version for *Scientific Reports*

Supplementary Information

Predictable Chronic Mild Stress during Adolescence Promotes Fear Memory Extinction in Adulthood

Jia-Hui Deng[1, 2, #], Wei Yan[1, 2, 3, #], Ying Han[1, *], Chen Chen[2], Shi-Qiu Meng[1], Cheng-Yu Sun[1, 2], Ling-Zhi Xu[1, 2], Yan-Xue Xue[1], Xue-Jiao Gao[1, 2], Na Chen[1], Fei-Long Zhang[2], Yu-Mei Wang[2, 5], Jie Shi[1, *], Lin Lu[1, 2, 4, *]

[1] National Institute on Drug Dependence and Beijing Key Laboratory of Drug Dependence, Peking University, Peking University, Beijing 100191, China

[2] Peking University Sixth Hospital, Peking University Institute of Mental Health, Key Laboratory of Mental Health, Ministry of Health (Peking University), National Clinical Research Center for Mental Disorders (Peking University Sixth Hospital), Peking University, Beijing 100191, China

[3] School of Basic Medical Sciences, Peking University Health Science Center, Beijing 100191, China

[4] Peking-Tsinghua Center for Life Sciences and PKU-IDG/McGovern Institute for Brain Research, Peking University, Beijing 100871, China

[5] Department of Mental Health, First Hospital of Hebei Medical University, Hebei Medical University, Shijiazhuang 050031, China

a.

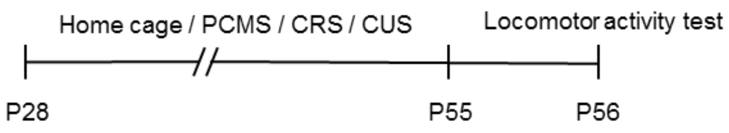

b.

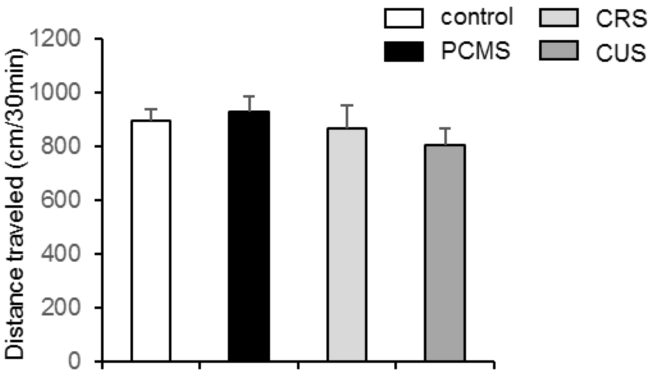

Supplementary Figure S1. Locomotor activities were not affected in PCMS, CRS or CUS rats. (**a**) Experimental timeline. (**b**) PCMS, CRS or CUS exposure during adolescence had no significant effect on locomotor activities (*n* = 8-10 per group). Data are expressed as mean ± SEM. PCMS, predictable chronic mild stress; CRS, chronic restraint stress; CUS, chronic unpredictable stress.

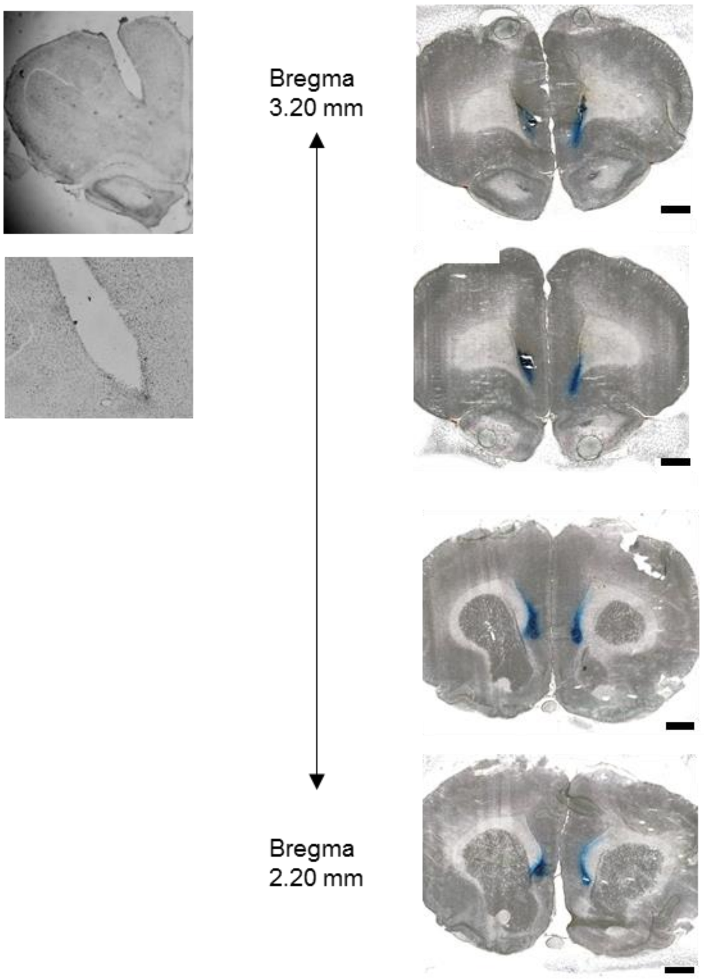

Supplementary Figure S2. Schematic representation and photomicrographs of cannula placements and Indian ink diffusion in the IL. The scale bar is 1 mm.

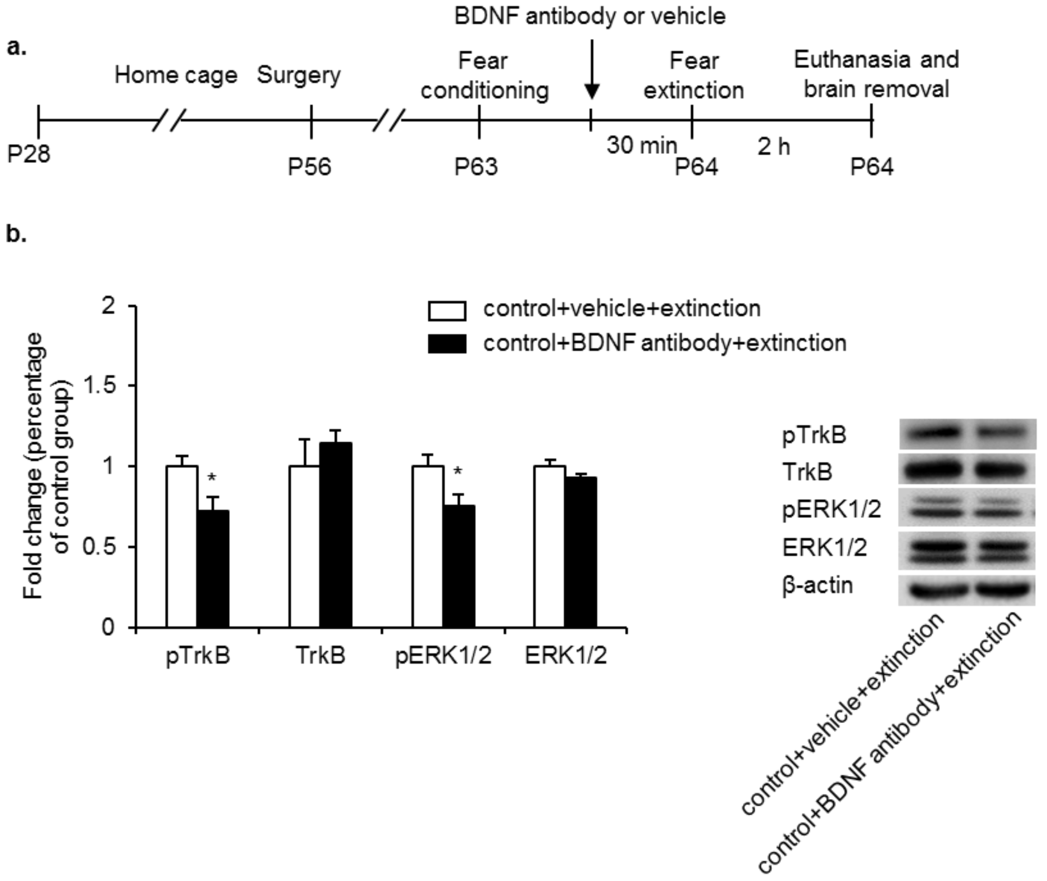

Supplementary Figure S3. Intra-IL infusion of BDNF antibody decreased levels of pTrkB and pERK1/2 in IL. (**a**) Experimental timeline. (**b**) Protein levels and representative western blot bands of pTrkB, TrkB, pERK1/2, and ERK1/2 in IL after BDNF antibody infusion. Blots shown are cropped from full-length. Data are expressed as mean ± SEM (n = 6 per group). *p < 0.05, compared with control+vehicle+extinction group.

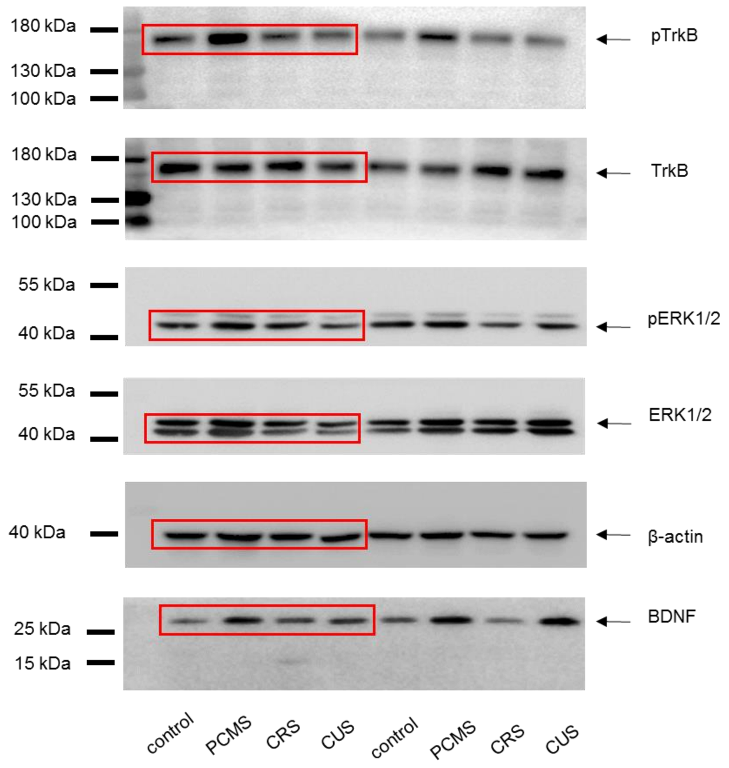

Supplementary Figure S4. Images of western blots provided in Fig. 2b in the main text.

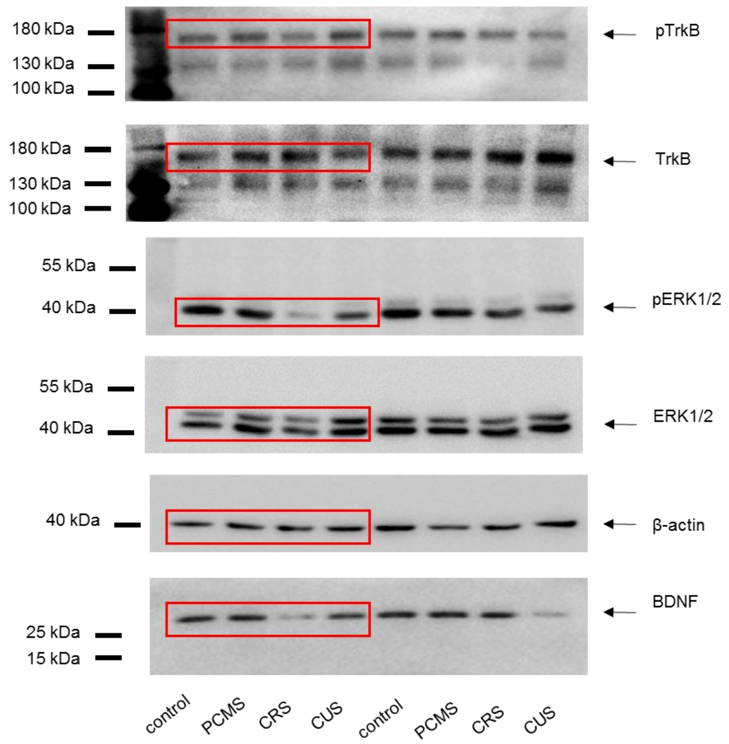

Supplementary Figure S5. Images of western blots provided in Fig. 2c in the main text.

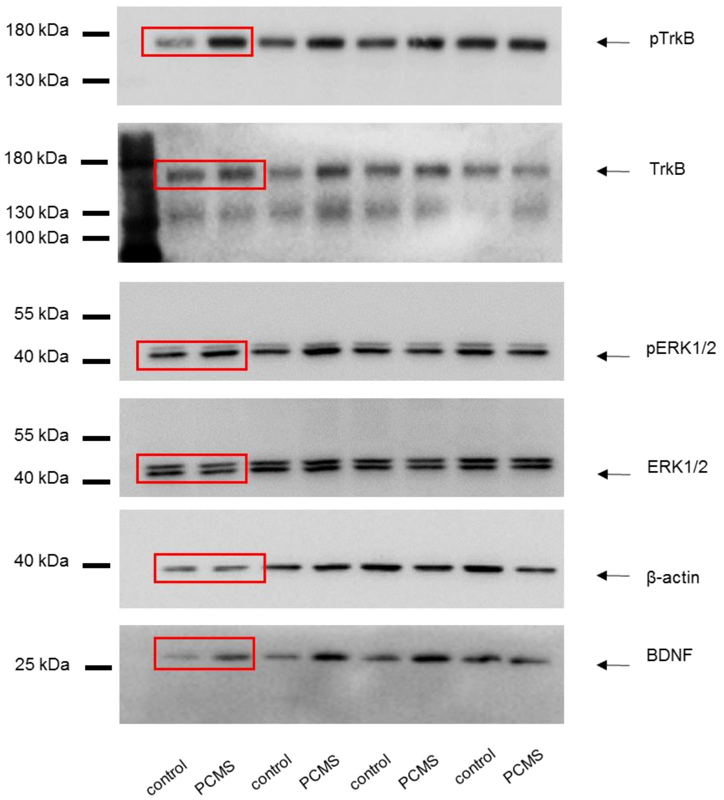

Supplementary Figure S6. Images of western blots provided in Fig. 3e in the main text.

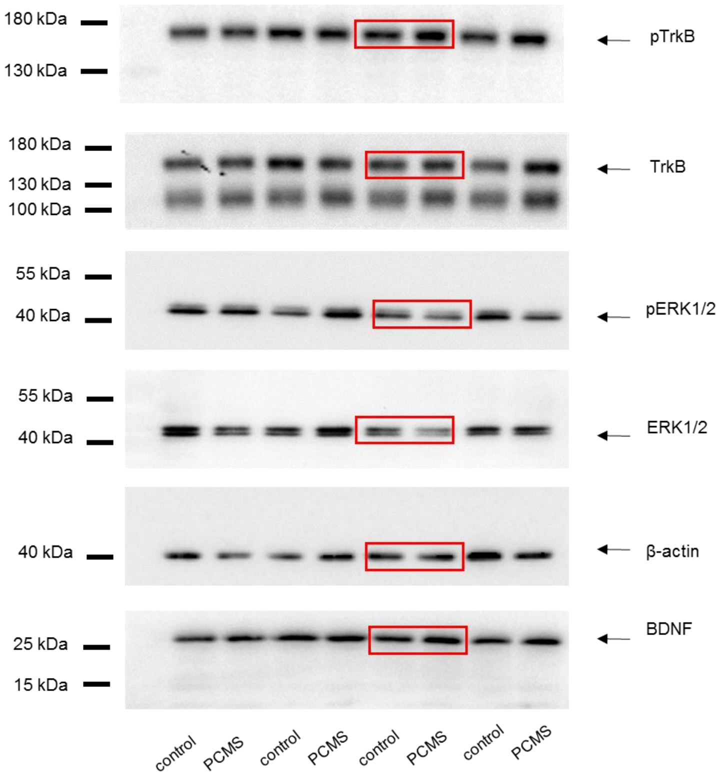

Supplementary Figure S7. Images of western blots provided in Fig. 3f in the main text.

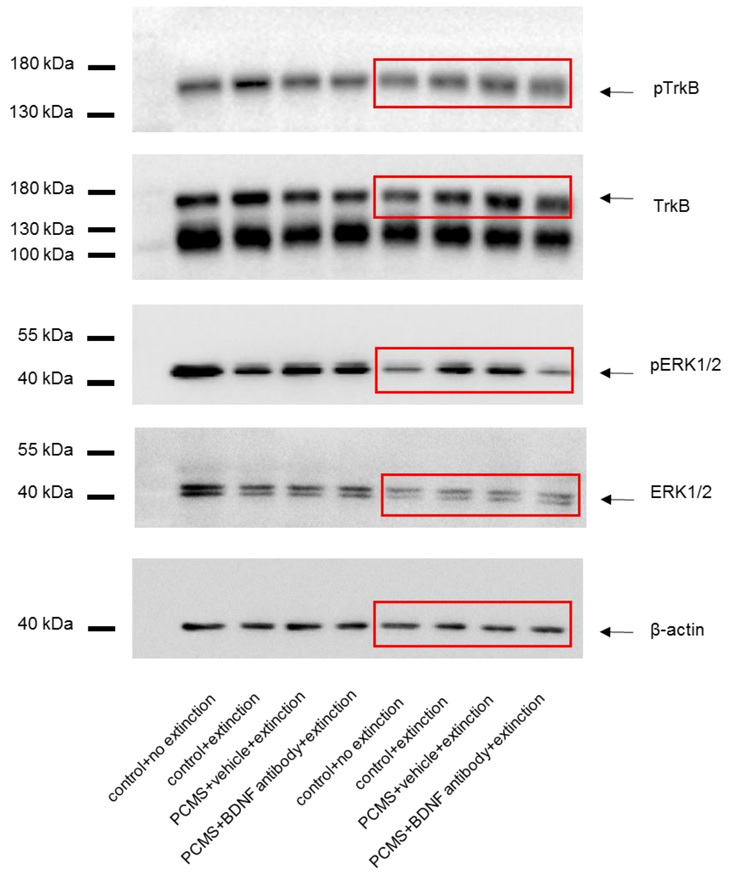

Supplementary Figure S8. Images of western blots provided in Fig. 4d in the main text.

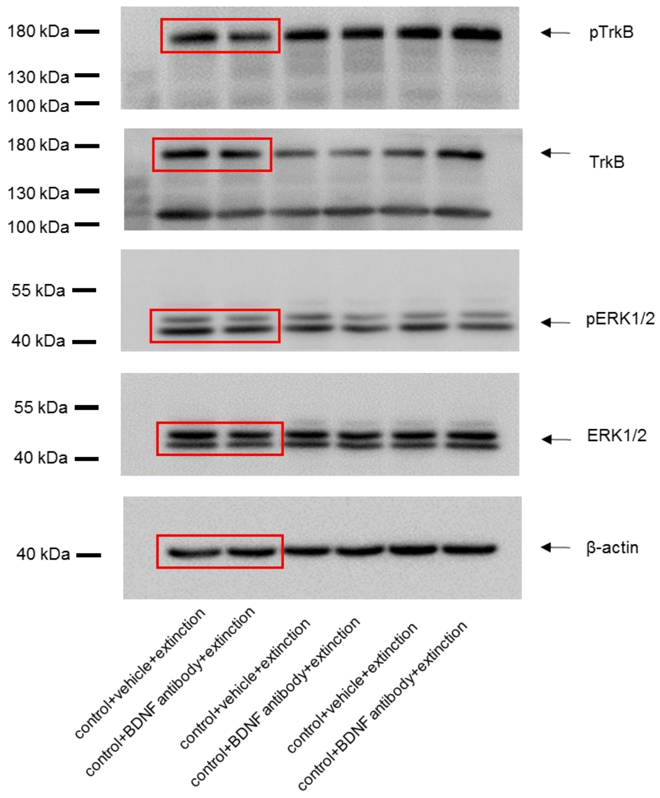

Supplementary Figure S9. Images of western blots provided in Supplementary Fig. S3b.
